# Intratracheal administration of adipose derived mesenchymal stem cells alleviates chronic asthma in a mouse model

**DOI:** 10.1186/s12890-018-0701-x

**Published:** 2018-08-08

**Authors:** Ranran Dai, Youchao Yu, Guofeng Yan, Xiaoxia Hou, Yingmeng Ni, Guochao Shi

**Affiliations:** 1 Department of Pulmonary and Critical Care Medicine, Ruijin Hospital, Shanghai Jiaotong University, School of Medicine, NO.197, Ruijin Er Road, Shanghai, 200025 China; 2 School of Medicine, Shanghai Jiaotong University, Shanghai, 200025 China

**Keywords:** Chronic asthma, Airway hyperresponsiveness, Mesenchymal stem cells, Regulatory T cells

## Abstract

**Background:**

Adipose-derived mesenchymal stem cell (ASCs) exerts immunomodulatory roles in asthma. However, the underlying mechanism remains unclear. The present study aimed to explore the effects and mechanisms of ASCs on chronic asthma using an ovalbumin (OVA)-sensitized asthmatic mouse model.

**Methods:**

Murine ASCs (mASCs) were isolated from male Balb/c mice and identified by the expression of surface markers using flow cytometry. The OVA-sensitized asthmatic mouse model was established and then animals were treated with the mASCs through intratracheal delivery. The therapy effects were assessed by measuring airway responsiveness, performing immuohistochemical analysis, and examining bronchoalveolar lavage fluid (BALF). Additionally, the expression of inflammatory cytokines and lgE was detected by CHIP and ELISA, respectively. The mRNA levels of serum indices were detected using qRT-PCR.

**Results:**

The mASCs grew by adherence with fibroblast-like morphology, and showed the positive expression of CD90, CD44, and CD29 as well as the negative expression of CD45 and CD34, indicating that the mASCs were successfully isolated. Administering mASCs to asthmatic model animals through intratracheal delivery reduced airway responsiveness, the number of lymphocytes (*P* < 0.01) and the expression of lgE (*P* < 0.01), IL-1β (*P* < 0.05), IL-4 (*P* < 0.001), and IL-17F (*P* < 0.001), as well as increased the serum levels of IL-10 and Foxp3, and the percentage of CD4 + CD25 + Foxp3+ Tregs in the spleen, and reduced the expression of IL-17 (*P* < 0.05) and RORγ.

**Conclusions:**

Intratracheal administration of mASCs alleviated airway inflammation, improved airway remodeling, and relieved airway hyperresponsiveness in an OVA-sensitized asthma model, which might be associated with the restoration of Th1/Th2 cell balance by mASCs.

## Background

As a common chronic respiratory disease, the prevalence of asthma keeps increases every year [1]. The characteristics of asthma include bronchial hyperreactivity and symptoms of airway obstruction [2]. The combination of corticosteroids and long-acting β_2_-adrenoceptor agonists is widely used to control asthma in most patients [3]. However, asthma is not yet cured and clinical symptoms are still difficult to alleviate [2]. Therefore, it is necessary to explore novel therapies and their mechanisms.

Multiple types of cells are involved in asthma pathology, including eosinophils, mast cells, T lymphocytes, invariant NKT cells, basophils, type 2 innate lymphoid cells (ILC2s) and others [4]. An imbalance of Th1 lymphocytes/Th2 lymphocytes (Th1/Th2) is thought to be involved in asthma pathogenesis of asthma [5], but this imbalance cannot fully explain the pathological mechanism of asthma. Regulatory T cells (Tregs), including natural regulatory T cells (nTregs) and inducible/adaptive regulatory T cells (iTregs or Tregs), play a more critical role in autoimmune diseases and allergic diseases through regulating helper T cells [6]. The phenotype of Tregs is characterized by constitutively high expression of cell surface molecules, such as CD25/GITR/CTLA4 and the transcription factor Foxp3 [5]. The role of Tregs in asthma has been demonstrated in both human and animal studies [7–9]. Shi et al. found that the Th2 response is associated with a deficiency of CD4 + CD25 + Tregs in patients with asthma [5]. Tao et al. revealed that Tregs are significantly less abundant in children with allergic rhinitis (AR) accompanyied by bronchial asthma (BA) than those with AR or BA alone or control subjects [10]. In a mouse asthma model of asthma, infusing CD4+ CD25+ Tregs can alleviate airway inflammation and airway hyperresponsiveness through increasing the serum level of IL-10 [9].

Mesenchymal stem cells (MSCs), pluripotent cells derived from early mesoderm, can differentiate into fat, bone, cartilage, epithelial cells and endothelial cells [11]. Many studies have reported that MSCs are beneficial to the remission of asthma remission [12–14]. It has been demonstrated that when MSCs are infused into mice with ovalbumin (OVA)-sensitized asthma, the serum IgE level is significantly downregulated and airway inflammation is markedly relieved [15, 16]. Importantly, adipose-derived mesenchymal stem cells (ASCs) reduce inflammation and induce Tregs in immune disorders such as rheumatoid arthritis [17]. However, the effects and mechanisms of ASCs in asthma have rarely been reported.

In the present study, mouse ASCs (mASCs) were intratracheally applied into mice in an OVA-sensitized chronic asthma model, and then the clinical symptoms of mouse asthma were extensively investigated. The underlying mechanisms were also revealed.

## Methods

### Animal preparation

The Ethics Committee of Shanghai Jiaotong University (Shanghai, China) approved this study. Ten male and eighty female Balb/c mice of 6–8 weeks age and 16–18 g weight were provided by Shanghai Silaike Laboratory Animal Co., Ltd. (Shanghai, China) and fed in the animal experimental center of school of Medicine, Shanghai Jiao Tong University at suitable environment. The male mice were fed with high fat diet for 4 weeks while the female mice were provided normal food and water under a 12 h/12 h light/dark cycle.

### Isolation and culture of murine ASCs

The male Balb/c mice were sacrificed via cervical dislocation and immersed into 75% alcohol for 2 min. Then, the groin subcutaneous fat tissues were aseptically removed and minced with scissors, followed by washing with phosphate buffer saline (PBS) and digestion with collagenase I [18]. The mASCs were obtained after the floating fat and undigested tissues were centrifuged. Next, the mASCs were resuspended in serum-free medium, inoculated into a T75 flask and cultured in a 37°C incubator with 5% CO_2_.

### Flow cytometry

The expression of CD90, CD44, CD29, CD34 and CD45 on mASCs was detected using flow cytometry (FACS, Epics Altra, Coulter, Beckman, USA). When the confluency of mASCs reached 80–90%, the cell density was adjusted to 1 × 10^6^/ml. Then the cell suspensions were acquired from mASCs of the mice, respectively. The FACS was conducted after incubation with antibodies against CD90-PE, CD44-PE, CD29-PE, CD34-PE, and CD45-PE (BD Pharmingen, San Diego, USA).

### Animal model, grouping and administration

Female Balb/c mice were randomly divided into four groups containing ten mice each group: PBS + PBS group, PBS + mASCs group, OVA + PBS group, and OVA+ mASCs group. An OVA-sensitized allergic asthma model was established following a previously described procedure [19]. Briefly, the mice in the PBS + PBS and PBS + mASCs groups were intraperitoneally injected with 100 μl PBS while the mice in the OVA + PBS and OVA + mASCs groups were intraperitoneally injected with 100 μl of 0.1% OVA (Sigma-Aldrich, Saint Louis, Missouri, USA) at Days 0, 7, and 14. Then, the mice were anesthetized by injecting 30 μl chloral hydrate and fixed in the operation panel. The mouse headswere lift, keeping an angle of 30° between the head and the ground. Mouse tongues were pulled out with wide-nose pliers, and the trace syringe was inserted into the trachea directly by the laryngeal glottis under laryngoscopy. In total, 30 μl of PBS were intratracheally injected into the mice of the PBS + PBS and OVA + PBS groups at Day 21 via endotracheal instillation. At the same time, 1 × 10^6^ mASCs in 30 μl PBS were intratracheally injected into the mice of the PBS + mASCs and OVA + mASCs groups. Thirty minutes after the transplantation, the mice were maintained in a chamber filled with 2.5% atomized-OVA for an extra 30 min. All the mice were challenged for 30 min with 2.5% atomized-OVA 3 times a week for 8 consecutive weeks. All female Balb/c mice were sacrificed within 24 h after the last atomized-OVA challenge through intraperitoneal injection of 300 mg/kg pentobarbital sodium.

### Airway hyperresponsiveness test

The mice were anesthetized by injecting 5 mg/kg midazolam and 100 mg/kg alcidione. Then airway responsiveness was determined with an EMMS system (EMMS, Hampshire, UK) by measuring lung resistance (R_L_) in response to inhaled methacholine (MCh; Sigma-Aldrich) at concentrations of 4 to 256 mg/mL for 20 s. The provocative challenge 100 (PC100) was calculated from the dose of MCh at which R_L_ was 100% above the baseline level.

### Preparation of bronchoalveolar lavage fluid

BALF was obtained by lavaging the trachea with cold PBS three times [20]. After centrifugation, the supernatants were used for cytokine analysis. The cell pellets were resuspended in PBS. The total number of cells was calculated under a microscope after Trypan blue staining, and the number of neutrophils, eosinophils, macrophages, and lymphocytes was determined after staining with Wright and Giemsa solutions.

### Immunohistochemical analysis

The left lung was fixed with 10% formalin solution for 24 h and embedded in paraffin. Then the samples were prepared using a standard protocol. The 5-μm-thick sections were subjected to HE, MASSON and AB-PAS staining according to previously described methods [21]. Besides, the expression of Muc5AC was visualized by immunohistochemical analysis after adding an anti-Muc5AC antibody (Abcam, Cambridge, UK).

### The percentage of CD4 + CD25 + Foxp3+ Tregs in spleen

The cells were filtrated after the spleen was fully ground and red blood cells were removed with erythrocyte lysis buffer. After washing twice with PBS, spleen cells were stained with anti-mouse CD4-FITC, CD25-APC, and Foxp3-PE antibodies (eBioscience, San Diego, CA, USA). The percentage of CD4 + CD25 + Foxp3+ Tregs was analyzed by flow cytometry (Epics Altra, Coulter, Beckman, USA).

### Quantitative real-time PCR (qRT-PCR) analysis

Total RNA of right lung tissues was extracted using Trizol (Invitrogen, Carlsbad, California, USA) and cDNA samples were prepared by a first strand cDNA synthesis kit (Promega, Wisconsin, Madison, USA) thereafter. The sequences of the primers were as follows: Foxp3, forward-5′-GCCTTCAGACGAGACTTG-3′, reverse − 5′-CATTGGGTTCTTGTCAGAG-3′; RORγt, forward-5′-CAGGAG CATGGAAGT-CGTC-3′, reverse-5′-CCGTGTAGAGGGCAATCTCA-3′; IL-10, forward-5′-CCCTT-TGCTATGGTGTCCT-3′, reverse-5′-GGATCTCCCTGGTTTCTCTT-3′; IL-17F, forward-5′-AGGGAAGAAGCAGCCATT-3′, reverse-5′-CCAACATCAACAGTAG-CAAA-3′; and β-actin, forward-5′-CAGAAGGACTCCTACGTG-3′, reverse-5′-GCTCGGTCAGGATCTTCATG-3′. β-actin was measured as an internal control. The relative expression of mRNA was normalized to expression of β-actin.

### Measurement of inflammatory cytokines in serum and BALF

Serum samples were obtained from mice in the four groups. Firstly, the mice were fixed in a supine position and the hair of the anterior chest was cut. After the skin was disinfected, 800 μl of blood was extracted from the area with the strongest heart beat using the cardiac puncture method. Then, the blood samples were centrifuged and the serum samples were obtained. Protein was extracted from the serum and BALF samples. In total, 50 μl of protein samples were acquired and normalized to the standard samples gradient elution on a slide. Fluorescein was measured as a detection signal. The expression of IL-1β, IL-4, IL-10 and IL-17F was quantified using CHIP by comparing the signal of these genes with those of the standard samples, respectively. Additionally, protein expression of IgE was measured by ELISA kits (eBioscience, San Diego, CA, USA) according to the manufacturer’s instructions.

### Statistical analysis

Data were analyzed by SPSS21.0 software and presented as mean ± standard deviation. A normality test and homogeneity variance analysis were first conducted to analyze the data. Then, the differences among multiple groups were determined using one-way ANOVA followed by the Fishers least significant difference (LSD) method. *P* < 0.05 was considered to be statistically significant.

## Results

### The morphological observation of mASCs and the expression of their surface markers

**Fig. 1:**
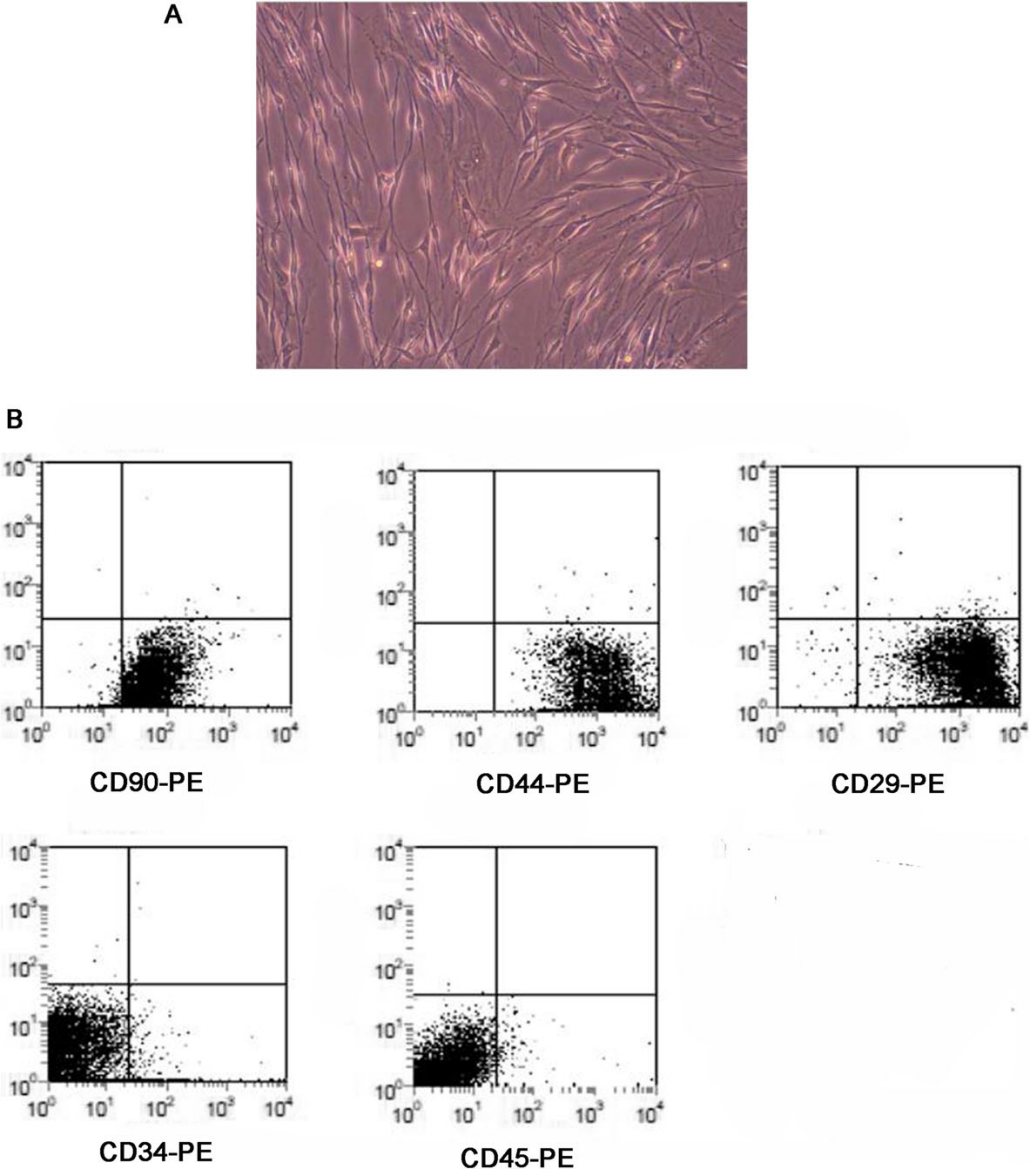
The identification of adipose-derived mesenchymal stem cells of mice (mASCs). **a** The morphology of mASCs under A microscope (400X); **b** The expression of CD90, CD44, CD29, CD34 and CD45 on the surface of mASCs by flow cytometry

The mASCs grew adherent with a fibroblast-like morphology, which gradually transformed to an irregular star shape with further passaging (Fig. 1a). FACS analysis showed that the surface expression of CD90, CD44, and CD29 on mASCs was high, while the expression of CD34 and CD45 was relatively low (Fig. 1b). These data demonstrated that mASCs isolation was successful.

### Effect of mASCs on the pathomorphology of lung tissue in OVA-induced asthma model mice

**Fig. 2:**
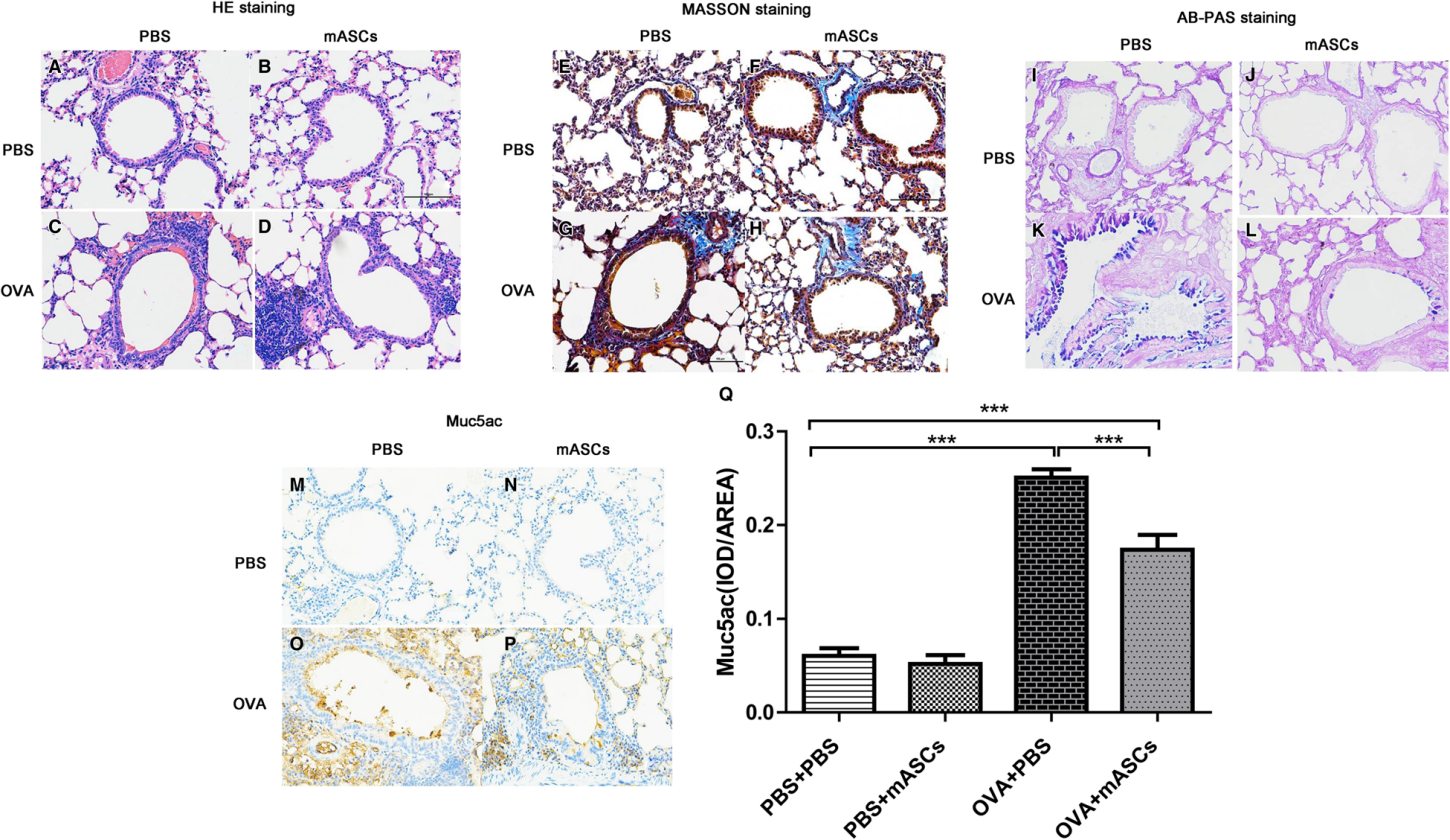
Applying mASCs improved the pathomorphology of lung tissues in OVA-induced asthma model mice. **a**-**d**, The HE staining of lung tissues in the PBS + PBS group, PBS + mASCs group, OVA + PBS group, and OVA+ mASCs group, respectively; **e**-**h** The fibrosis of lung tissues in the PBS + PBS group, PBS + mASCs group, OVA + PBS group, and OVA+ mASCs group, respectively, by MASSON staining; **i**-**l** The mucus of lung tissues in the PBS + PBS group, PBS + mASCs group, OVA + PBS group, and OVA+ mASCs group, respectively, by AB-PAS staining; **m**-**p** The Muc5ac expression of lung tissues in the PBS + PBS group, PBS + mASCs group, OVA + PBS group, and OVA+ mASCs group, respectively, by immunohistochemical analysis. Bar = 100 μm. mASCs, mouse adipose-derived mesenchymal stem cells; PBS, phosphate buffered saline; OVA, ovalbumin. **q** The quantitative results of Muc5ac in figure 2m-p

Compared to control mice, OVA-challenged mice showed abnormal lung structure by HE staining, including inverted bronchial mucosa cilia, wall thickening, and swelled mucous membrane, while treatment with mASCs in the OVA group markedly reduced the number of inflammatory cells (Fig. 2a-d). To determine differences in tissue composition of the experimental groups, MASSON staining was conducted. The results found that the manifestation of the tissues in the four groups was not significantly different, and fibrosis was not observed (Fig. 2e-f). In addition, the tissues of mice in the OVA + mASCs group had only slight fibrosis, indicating that the transplantating mASCs could relieve fibrosis in the mice with chronic asthma (Fig. 2g-h). According to the AB-PAS staining, the tissues of mice in the PBS + PBS and PBS + mASCs groups were not significantly different. No mucus in the lumen and no goblet cell hyperplasia were observed in the PBS + PBS and PBS + mASCs groups (Fig. 2i-j). However, mice in the OVA+ PBS group exhibited goblet cell hyperplasia and increased blue mucus, but goblet cell hyperplasia was relieved and blue mucus was reduced in the OVA + mASCs group (Fig. 2k-l). Furthermore, immunohistochemical analysis indicated that Muc5ac secretion was low and similar in the PBS + PBS and PBS + mASCs groups (Fig. 2m-n), but was high in the OVA + PBS group. At the same time, in the OVA + mASCs group, the secretion of Muc5ac protein was obviously reduced compared with the level in the OVA + PBS group (Fig. 2o-p, *P* < 0.001). The quantitative results of Muc5ac are shown in Fig. 2q.

### Effect of mASCs on lung function and airway hyperresponsiveness in OVA-induced asthma model mice

**Fig. 3:**
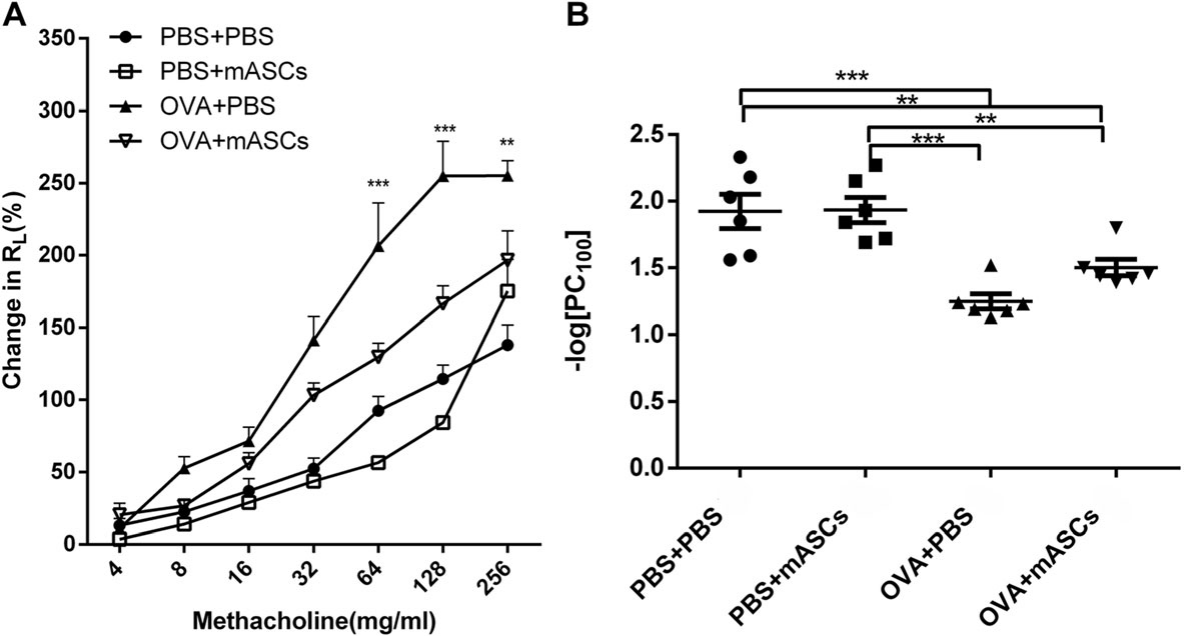
Applying mASCs improved lung function and airway hyperresponsiveness in OVA-induced asthma model mice. **a** The lung resistance (R_L_) in response to inhaled methacholine by airway responsiveness test; **b** The provocative challenge 100 (PC100) by airway responsiveness test. **P < 0.01, and ***P < 0.001. mASCs, mouse adipose-derived mesenchymal stem cells; PBS, phosphate buffer saline; OVA, ovalbumin

A pulmonary function test was conducted to evaluate whether the application of mASCs could restore lung function to some extent. For normal mice, changes to R_L_ in mASCs-treated mice were not significantly different from that of PBS-treated mice (Fig. 3a). However, the R_L_ in OVA-challenged mice kept increasing as methacholine concentration increased, indicating that the asthma model was established successfully. After intratracheal administration of mASCs, changes to R_L_ in the asthma model significantly declined when methacholine concentration was set to 64 mg/mL, 128 mg/mL and 256 mg/mL, respectively (*P* < 0.01, Fig. 3a). Further analysis of –log [PC100] among all these four groups showed that the inhalation of OVA significantly increased airway resistance while intratracheal transplantation mASCs could reduce airway responsiveness (*P* < 0.01, Fig. 3b).

### Effect of mASCs on the percentage of CD4 + CD25 + Foxp3 + Tregs in spleen in OVA-induced asthma model mice

**Fig. 4:**
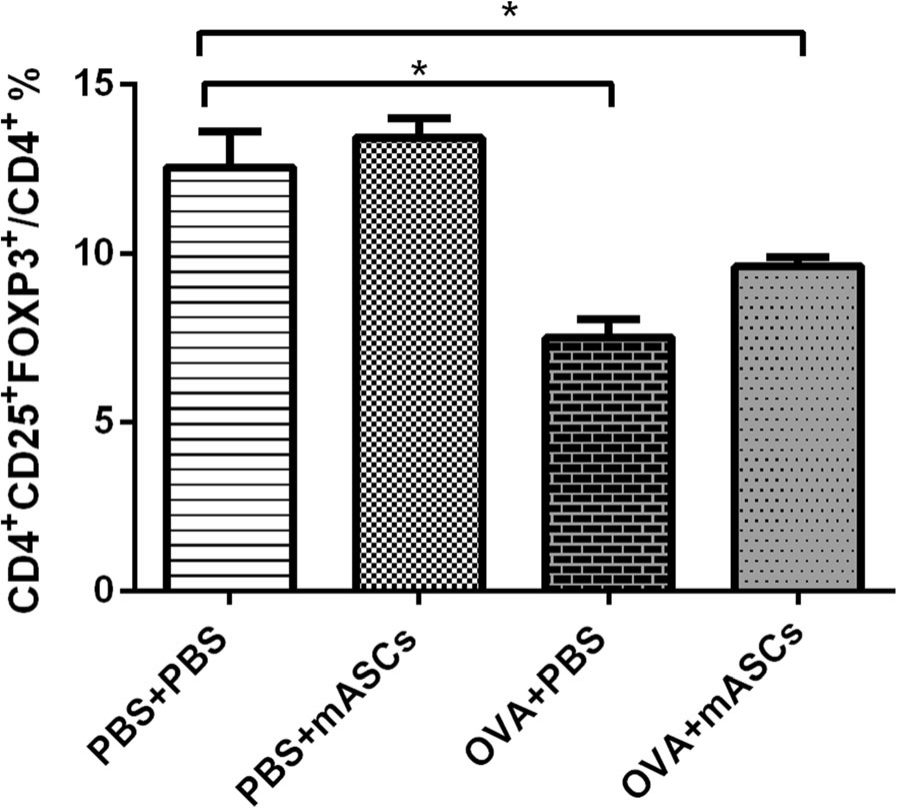
The percentage of CD4+ CD25+ Foxp3+ Tregs in spleen in the PBS + PBS group, PBS + mASCs group, OVA + PBS group, and OVA+ mASCs group, respectively, by flow cytometry analysis. *P < 0.05. mASCs, mouse adipose-derived mesenchymal stem cells; PBS, phosphate buffered saline; OVA, ovalbumin

The percentage of CD4 + CD25 + Foxp3 + Tregs in spleen of control mice was similar regardless of mASCs while the percent of Tregs significantly decreased in OVA-challenged mice compared with control mice (*P* < 0.05, Fig. 4). Although the transplantation of mASCs through trachea in an asthma model promoted the percentage of Tregs in the spleen, the difference was not statistically significant (Fig. 4).

### Effect of mASCs on cell count and subsets in BALF in OVA-induced asthma model mice

**Fig. 5:**
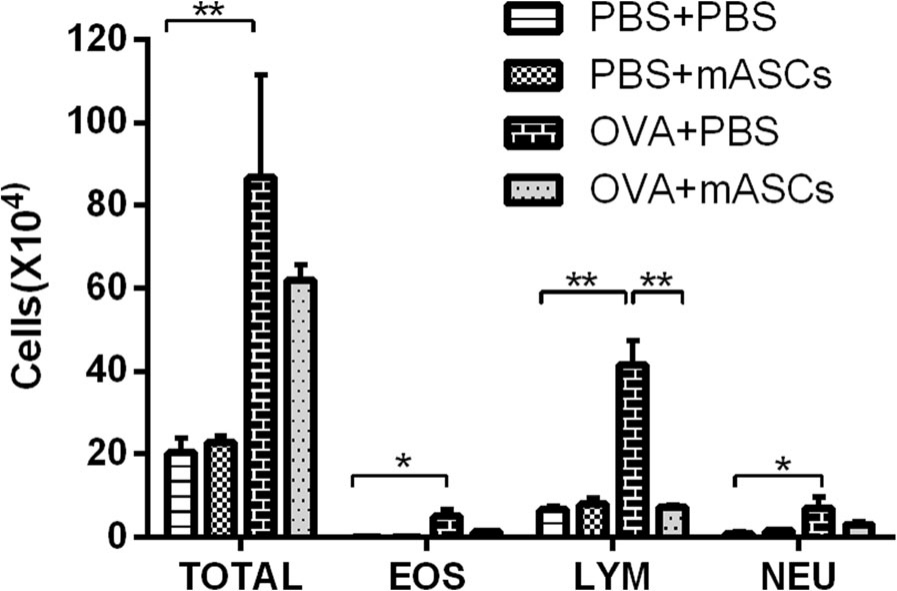
Cell counts and subsets in bronchoalveolar lavage fluid (BALF) samples in the PBS + PBS group, PBS + mASCs group, OVA + PBS group, and OVA+ mASCs group, respectively. *P < 0.05 and **P < 0.01. mASCs, mouse adipose-derived mesenchymal stem cells; PBS, phosphate buffered saline; OVA, ovalbumin

In the control mice, the administration of mASCs did not change the total cell number in BALF nor the cell subsets. However, the total cell number and lymphocytes were significantly higher in asthma model mice than that in control mice (*P* < 0.01, Fig. 5). In addition, eosinophils and neutrophils were significantly higher in asthma model mice than that in control mice (*P* < 0.05, Fig. 5). Compared with mice exposed to OVA challenge alone, applying mASCs in OVA-challenged mice reduced the number of cell subsets, eosinophils, neutrophils, and lymphocytes, while only the alteration of lymphocytes was statistically different (*P* < 0.01, Fig. 5).

### Effect of mASCs on inflammatory cytokines in OVA-induced asthma model mice

**Fig. 6:**
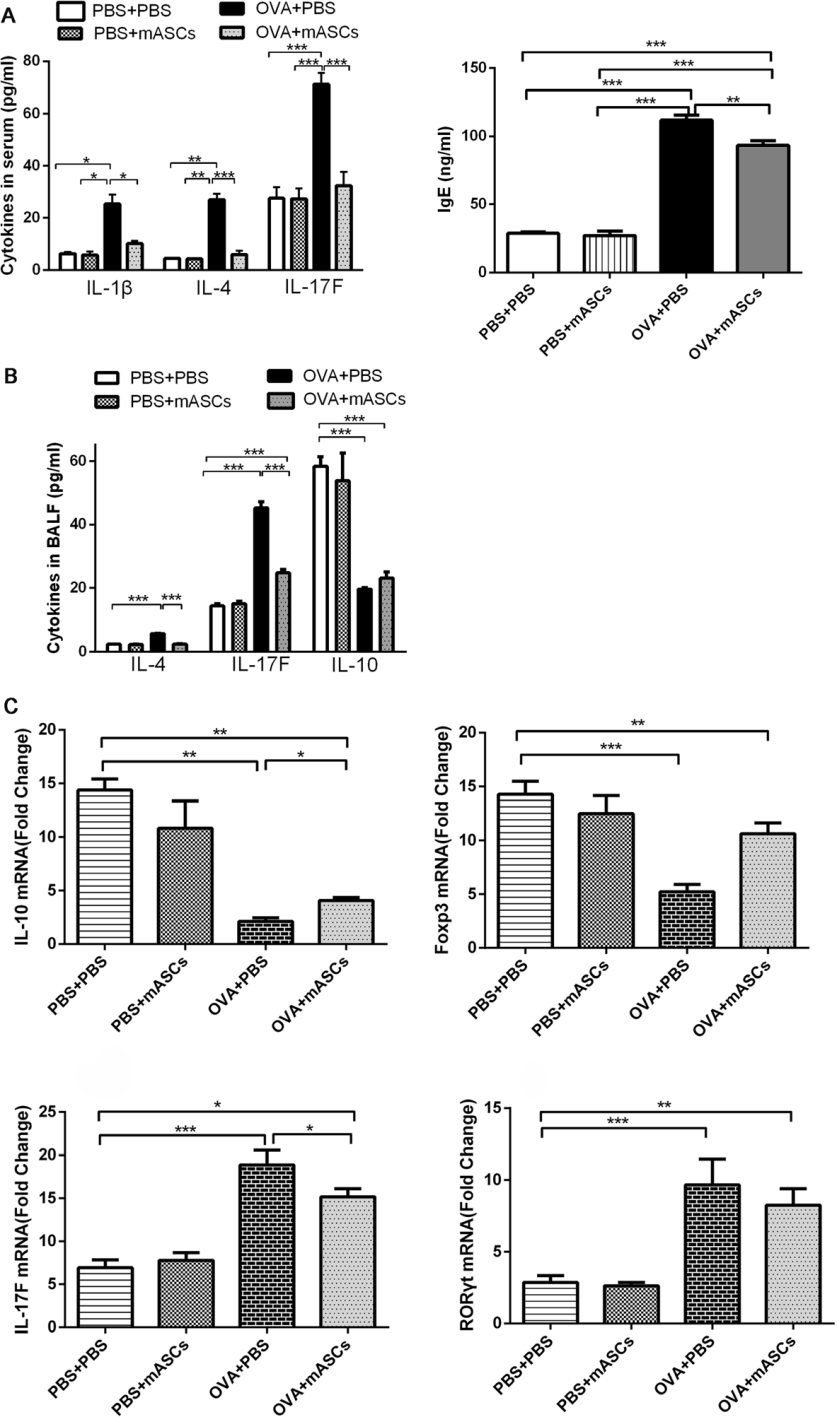
Applying mASCs improved the inflammation in OVA-induced asthma model mice. **a** The expression of serum IL-1β, IL-4, IL-17F, and lgE in the PBS + PBS group, PBS + mASCs group, OVA + PBS group, and OVA+ mASCs group, respectively, by CHIP and ELISA; **b** The expression of IL-4, IL-17F, and IL-10 in BALF in the PBS + PBS group, PBS + mASCs group, OVA + PBS group, and OVA+ mASCs group, respectively, by CHIP; **c** The mRNA levels of IL-10, Foxp3, IL-17, and RORγ in the lung tissues of PBS + PBS group, PBS + mASCs group, OVA + PBS group, and OVA+ mASCs group, respectively, by qRT-PCR. *P < 0.05, **P < 0.01 and ***P < 0.001. mASCs, mouse adipose-derived mesenchymal stem cells; PBS, phosphate buffered saline; OVA, ovalbumin; BALF, bronchoalveolar lavage fluid

The expression of serum IL-1β, IL-4, and IL-17F were all significantly increased in the asthma model animals when compared with levels in mice from the PBS + PBS group (*P* < 0.05, *P* < 0.01, and *P* < 0.001, respectively, Fig. 6a). However, the secretion of all these three cytokines drastically decreased after intratracheal transplantation of mASCs when compared with those in the asthma model group (*P* < 0.05, *P* < 0.001, and *P* < 0.001, respectively, Fig. 6a). The expression of serum IgE was determined by ELISA, and the results showed that IgE level in OVA-sensitized mice was dramatically elevated when compared with the level in the PBS + PBS group (*P* < 0.01, Fig. 6a). However, intratracheal administration of mASCs in OVA challenge mice could inhibit serum IgE level, which was significantly different when compared with OVA + PBS group (*P* < 0.01, Fig. 6a). A similar trend was found in the BALF level of both IL-4 and IL17F, which were significantly enhanced after OVA-challenged (all *P* < 0.001, Fig. 6b) but decreased after administration of mASCs (all *P* < 0.001, respectively, Fig. 6b). However, the level of IL-10 decreased OVA challenge but increased after administration of mASCs (*P* < 0.05). In addition, in the lung tissues, the mRNA levels of IL-10 and Foxp3 in OVA-treated mice was significantly reduced (*P* < 0.01 and *P* < 0.001, Fig. 6c), while the expression of IL-17 and RORγ was increased compared with expression in the PBS + PBS group (all *P* < 0.001, Fig. 6c).

## Discussion

Asthma is an airway chronic inflammatory disease of the airway involving a variety of cells and components. This chronic inflammation is associated with airway hyperresponsiveness. The mouse asthma model used in the present study perfectly mimicked the pathophysiological characteristics of human asthma. The results demonstrated that intratracheal administration of mASCs could not only reduce airway hyperresponsiveness, inflammatory cell infiltration, and Muc5ac secretion, but also improve airway remodeling.

Th1/Th2 cell imbalance is involved in asthma pathogenesis [22]. Th1 cells exert a protective effect on asthma through releasing IFN-γ and IL-2, while Th2 cells mainly secrete IL-4, IL-5, and IL-13, and then promote the development of asthma [23, 24]. IL-4 can promote the secretion of IgE and increase the secretion of Muc5ac protein in the airway, which leads to high airway response [23, 24]. In this study, we found that Th1 cell secretion was insufficient while Th2 cell secretion was excessive in a chronic asthma mouse model. Furthermore, the expression of serum IL-1β, IL-4 and IL-17F was higher in the asthmatic model compared to those being transplanted by mASCs, indicating that cell therapy might improve the degree of asthma airway inflammation and airway remodeling through regulating the Th1/Th2 ratio. Our results were similar with those of a study by Nemeth [25].

The malfunction of Tregs is another important mechanism of asthma. Growing evidences shows that asthma is closely related to Tregs [26]. The decline in the number of Tregs as well as their function in asthma is an important manifestation of an immune disorders [27]. A previous study showed that human and mouse MSCs could be induced into CD4 + CD25+ Tregs both in vitro and in vivo, and the immune regulation mediated by MSCs was associated with an increase in number of T cells and the associated cytokine levels [28]. In the present study, the ratio of Tregs to CD4+ T lymphocytes in spleen of asthma model animals was significantly lower than that of the normal group, while the administration of mASCs improved the percentage of Tregs, and reduced the degree of infiltration of inflammatory cells in small bronchial and vascular submucosal tissues in chronic asthmatic mice. Importantly, the transplantation of mASCs showed no influence on normal lung tissue.

As pro-inflammatory cells, Th17 cells can induce the occurrence of asthma and autoimmune diseases by secreting inflammatory factors such as IL-17A, IL-17F, IL-6 and tumor necrosis factor-α [29]. RORγt is the key transcription factor regulating Th17 cell differentiation [30]. In the present study, the IL-17F level in serum and BALF was higher in asthmatic model than that in normal mice, while the IL-10 level was lower in asthmatic model animals. Applying mASCs could downregulate IL-17 and RORγ in asthmatic mice, and upregulate IL-10 and FOXP3, implying that cell therapy could restore the balance of Th17/Treg cells through suppressing the differentiation of Th17 cells and inducing the transcription of Foxp3, which ultimately alleviated airway inflammation and remodeling.

## Conclusions

In conclusion, intratracheal administration of mASCs could alleviate the airway inflammation of lung tissue in an OVA-sensitized mouse asthma model, improve airway remodeling and relieve airway hyperresponsiveness. The mechanism might be associated with the restoration of Th1/Th2 cell balance by mASCs.

## Abbreviations

ASCs: Adipose-derived mesenchymal stem cell
BALF: Bronchoalveolar lavage fluid
LSD: Least significant difference
MSCs: Mesenchymal stem cells
OVA: Ovalbumin
PBS: Phosphate buffer saline
TH1/TH2: Th1 lymphocytes/Th2 lymphocytes

## References

[1] Barnes PJ (2010). New therapies for asthma: is there any progress?. Trends Pharmacol Sci.

[2] Braman SS (2006). The global burden of asthma. Chest J.

[3] Chung KF, Adcock IM (2004). Combination therapy of long-acting β2-adrenoceptor agonists and corticosteroids for asthma. Treat Respir Med.

[4] Barnes PJ (2008). Immunology of asthma and chronic obstructive pulmonary disease. Nat Rev Immunol.

[5] Shi Y, Shi G, Wan H, Jiang L, Ai X, Zhu H, Tang W, Ma J, Jin X, Zhang B (2011). Coexistence of Th1/Th2 and Th17/Treg imbalances in patients with allergic asthma. Chin Med J.

[6] Lloyd CM, Hawrylowicz CM (2009). Regulatory T cells in asthma. Immunity.

[7] Kearley J, Barker JE, Robinson DS, Lloyd CM (2005). Resolution of airway inflammation and hyperreactivity after in vivo transfer of CD4+ CD25+ regulatory T cells is interleukin 10 dependent. J Exp Med.

[8] Lewkowich IP, Herman NS, Schleifer KW, Dance MP, Chen BL, Dienger KM, Sproles AA, Shah JS, Köhl J, Belkaid Y (2005). CD4+ CD25+ T cells protect against experimentally induced asthma and alter pulmonary dendritic cell phenotype and function. J Exp Med.

[9] Boudousquie C, Pellaton C, Barbier N, Spertini F (2009). CD4+ CD25+ T cell depletion impairs tolerance induction in a murine model of asthma. Clin Exp Allergy.

[10] Tao B, Ruan G, Wang D, Li Y, Wang Z, Yin G (2015). Imbalance of peripheral Th17 and regulatory T Cells in children with allergic Rhinitis AND bronchial asthma. Iran J Allergy Asthma Immunol.

[11] Charbord P (2010). Bone marrow mesenchymal stem cells: historical overview and concepts. Hum Gene Ther.

[12] Ge X, Bai C, Yang J, Lou G, Li Q, Chen R (2013). Effect of mesenchymal stem cells on inhibiting airway remodeling and airway inflammation in chronic asthma. J Cell Biochem.

[13] Mathias LJ, Khong SM, Spyroglou L, Payne NL, Siatskas C, Thorburn AN, Boyd RL, Heng TS (2013). Alveolar macrophages are critical for the inhibition of allergic asthma by mesenchymal stromal cells. J Immunol.

[14] Ou-Yang H-F, Huang Y, Hu X-B, Wu C-G (2011). Suppression of allergic airway inflammation in a mouse model of asthma by exogenous mesenchymal stem cells. Exp Biol Med.

[15] Sun YQ, Deng MX, He J, Zeng QX, Wen W, Wong DS, Tse HF, Xu G, Lian Q, Shi J (2012). Human pluripotent stem cell-derived mesenchymal stem cells prevent allergic airway inflammation in mice. Stem Cells.

[16] Temelkovski J, Hogan SP, Shepherd DP, Foster PS, Kumar RK (1998). An improved murine model of asthma: selective airway inflammation, epithelial lesions and increased methacholine responsiveness following chronic exposure to aerosolised allergen. Thorax.

[17] Gonzalez-Rey E, Gonzalez MA, Varela N, O'Valle F, Hernandez-Cortes P, Rico L, Büscher D, Delgado M (2010). Human adipose-derived mesenchymal stem cells reduce inflammatory and T cell responses and induce regulatory T cells in vitro in rheumatoid arthritis. Ann Rheum Dis.

[18] Cho KS, Park HK, Park HY, Jung JS, Jeon SG, Kim YK, Roh HJ (2009). IFATS collection: immunomodulatory effects of adipose tissue-derived stem cells in an allergic rhinitis mouse model. Stem Cells.

[19] Kianmeher M, Ghorani V, Boskabady MH (2016). Animal model of asthma, various methods and measured parameters: a methodological review. Iran J Allergy Asthma Immunol.

[20] Feizpour A, Boskabady MH, Ghorbani A (2014). Adipose-derived stromal cell therapy affects lung inflammation and tracheal responsiveness in Guinea pig model of COPD. PLoS One.

[21] Mohammadian M, Boskabady MH, Kashani IR, Jahromi GP, Omidi A, Nejad AK, Khamse S, Sadeghipour HR (2016). Effect of bone marrow derived mesenchymal stem cells on lung pathology and inflammation in ovalbumin-induced asthma in mouse. Iran J Basic Med Sci.

[22] Mazzarella G, Bianco A, Catena E, De Palma R, Abbate G (2000). Th1/Th2 lymphocyte polarization in asthma. Allergy.

[23] H-j P, Lee C-M, Jung ID, Lee JS, Y-i J, Chang JH, Chun S-H, Kim M-J, Choi I-W, Ahn S-C (2009). Quercetin regulates Th1/Th2 balance in a murine model of asthma. Int Immunopharmacol.

[24] Kidd P (2003). Th1/Th2 balance: the hypothesis, its limitations, and implications for health and disease. Altern Med Rev.

[25] Nemeth K, Keane-Myers A, Brown JM, Metcalfe DD, Gorham JD, Bundoc VG, Hodges MG, Jelinek I, Madala S, Karpati S (2010). Bone marrow stromal cells use TGF-β to suppress allergic responses in a mouse model of ragweed-induced asthma. Proc Natl Acad Sci.

[26] Yun L, Xin-sheng F, Jing-hua Y, Li X, Shan-shan W (2014). CD4+ CD25+ FOXP3+ T cells, Foxp3 gene and protein expression contribute to antiasthmatic effects of San’ao decoction in mice model of asthma. Phytomedicine.

[27] Poon AH, Chouiali F, Tse SM, Litonjua AA, Hussain SN, Baglole CJ, Eidelman DH, Olivenstein R, Martin JG, Weiss ST (2012). Genetic and histological evidence for autophagy in asthma pathogenesis. J Allergy Clin Immunol.

[28] Svobodova E, Krulova M, Zajicova A, Pokorna K, Prochazkova J, Trosan P, Holan V (2011). The role of mouse mesenchymal stem cells in differentiation of naive T-cells into anti-inflammatory regulatory T-cell or proinflammatory helper T-cell 17 population. Stem Cells Dev.

[29] Yang J, Sundrud MS, Skepner J, Yamagata T (2014). Targeting Th17 cells in autoimmune diseases. Trends Pharmacol Sci.

[30] Yang XO, Pappu BP, Nurieva R, Akimzhanov A, Kang HS, Chung Y, Ma L, Shah B, Panopoulos AD, Schluns KS (2008). T helper 17 lineage differentiation is programmed by orphan nuclear receptors RORα and RORγ. Immunity.

